# Advances in Research on the Improvement of Low-Salt Meat Product Through Ultrasound Technology: Quality, Myofibrillar Proteins, and Gelation Properties

**DOI:** 10.3390/molecules29204926

**Published:** 2024-10-17

**Authors:** Xiuyun Guo, Shuangyi Xu, Chao Fu, Zengqi Peng

**Affiliations:** 1 School of Tourism and Cuisine, Yangzhou University, Yangzhou 225127, China; mx120221251@stu.yzu.edu.cn (S.X.); mx120231286@stu.yzu.edu.cn (C.F.); 2 Key Laboratory of Chinese Cuisine Intangible Cultural Heritage Technology Inheritance, Ministry of Culture and Tourism, Yangzhou 225127, China; 3 College of Food Science and Technology, National Center of Meat Quality and Safety Control, Nanjing Agricultural University, Nanjing 210095, China

**Keywords:** ultrasound, salt reduction, processed meat, flavor, water holding capacity

## Abstract

The high sodium content in meat products poses health risks to consumers and does not align with modern green and healthy living standards. Current strategies for directly reducing the sodium content in meat products are limited by their negative impact on the sensory or quality attributes of the products. In recent years, there has been great interest in applying ultrasound technology to reduce sodium content. This paper discusses the advantages and disadvantages of current mainstream strategies for reducing the sodium content in meat products, as well as the potential mechanisms by which ultrasound-assisted marination improves the quality of low-salt meat products. The main findings indicate that ultrasound, through its cavitation and mechanical effects, facilitates the transition of proteins from stable insoluble aggregates to stable soluble complexes, exposing more hydrophilic groups and, thus, enhancing protein solubility. At the same time, ultrasound promotes a greater number of proteins to participate in the formation of interfacial layers, thereby increasing emulsifying activity. Furthermore, ultrasound treatment promotes the interaction between proteins and water, leading to partial unfolding of protein chains, which allows polar residues to more readily capture water in the gel, thereby improving the water-holding capacity of the gel. These effects will contribute to the formation of high-quality low-salt meat products. However, variations in the frequency, intensity, and duration of ultrasound treatment can lead to differing effects on the quality improvement of low-salt meat products.

## 1. Introduction

Saltiness, as the “chief of all flavors”, is a fundamental taste in most dishes and is indispensable in the processing of meat products [1]. Salt, being the primary source of saltiness, plays a crucial role in culinary preparations and food processing [2]. However, with the development of the socio-economic environment and changes in people’s daily lifestyles, there has been an increase in mental activity and a decrease in physical activity, leading to a reduced rate of salt metabolism in the body. Concurrently, there is an increased demand for industrially processed foods, dining out, and food delivery services, resulting in a paradox where the body’s need for salt decreases, but salt intake rises [3]. Meat products typically contain substantial amounts of sodium chloride. High-salt meat products such as ham, sausages, dry-cured meats, and preserved meat are particularly favored by consumers due to their unique sensory characteristics, accounting for approximately 20% of total sodium intake [2]. Excessive sodium intake has been proven to increase various health risks, including hypertension, cardiovascular diseases, cancer, and other chronic conditions [4,5]. Over the past few decades, food manufacturers worldwide have been required to reduce the salt content in many food products to meet certain standards [6]. Direct salt reduction, achieved by lowering the amount of salt added to food, is a direct, effective, and convenient method for salt reduction. However, reducing the amount of sodium salt directly often comes at the expense of sacrificing the taste and quality of food, which may not be accepted by most consumers [7,8]. This has driven the food industry to urgently seek new technologies or methods to reduce sodium content in foods.

Ultrasonic technology, as a novel non-thermal processing technique, has been widely employed in food processing and manufacturing due to its beneficial effects on meat and meat products through cavitation effects and mechanical action [9,10]. Ultrasonic waves can be categorized into low-intensity ultrasound (LIU, frequency > 100 kHz, intensity < 1 W/cm^2^) and high-intensity ultrasound (HIU, frequency: 20–100 kHz, intensity > 1 W/cm^2^) based on their frequencies [11,12]. The 20–40 kHz ultrasonic method finds wider application in the food industry [12,13]. When ultrasound technology is applied to the production and processing of meat products, it helps to shorten curing times, promote uniform salt distribution, and reduce salt usage [14,15]. This is achieved through intense vibrations and cavitation effects that activate the brine and disrupt the muscle fiber structure, facilitating the transfer of substances and improving the absorption rate of NaCl from the brine into the muscle [11,16]. On the other hand, ultrasound technology can also impart better tenderness and water-holding capacity to meat products [9,17]. By utilizing its cavitation and mechanical effects, ultrasound encourages proteins to transition from stable insoluble aggregates to stable soluble complexes, exposing more hydrophilic groups and, thus, enhancing protein solubility [10,18]. Additionally, ultrasound facilitates more proteins participating in the formation of interfacial layers, which increases emulsifying activity [19]. Furthermore, ultrasound treatment promotes interactions between proteins and water, leading to partial unfolding of protein chains, making it easier for polar residues to capture moisture in the gel, and, thereby, improving the water-holding capacity of the gel [20,21]. These effects contribute to the formation of high-quality low-salt meat products. Therefore, in novel salt reduction techniques, ultrasonic treatment has become one of the most suitable and popular methods due to its efficiency, relatively low cost, non-thermal nature, and environmental friendliness [11]. However, some current studies have different findings, suggesting that the application of ultrasonic technology in the processing of low-salt meat products can have adverse effects on their quality [22,23,24,25]. The main reason for this is that variations in the frequency, intensity, and duration of ultrasonic treatment can lead to different impacts on the quality of various types of low-salt meat products. In conclusion, existing publications in this area lack an across-the-board systematic summary of the effect and mechanism of the ultrasonic technology in terms of their effects on meat quality, myofibrillar proteins, and gelation properties at a low salt concentration.

Research indicates that Asian countries have the highest salt intake, with daily consumption exceeding 12 g, which significantly surpasses the maximum intake recommended by the World Health Organization (salt not exceeding 5 g/day and sodium not exceeding 2 g/day) [26]. With the proposal of the “Healthy China 2030” plan in China and the increasing awareness of health and improvement in living standards among residents, there has been a growing demand for green and healthy food products. Therefore, the application of ultrasonic technology to reduce sodium chloride content in food and meat products has attracted sustained attention from researchers and the food industry.

Therefore, this article discusses feasible technologies and methods for reducing sodium content in meat products while addressing the challenges and limitations faced. Furthermore, the present paper is aimed to summarize the effect of ultrasound technology and discuss the potential underlying mechanisms that affect the quality of low-salt meat and meat products from the perspective of protein and gel properties. Finally, the challenges of ultrasonic technology in the processing and production of low-salt meat and meat products are discussed. The aim is to consolidate existing findings to provide a more comprehensive theoretical basis for the subsequent research and practical application of ultrasonic technology in the marination and salt reduction of meat products.

## 2. Current Research Status of Low-Salt Meat Product Processing Technologies

**Figure 1:**
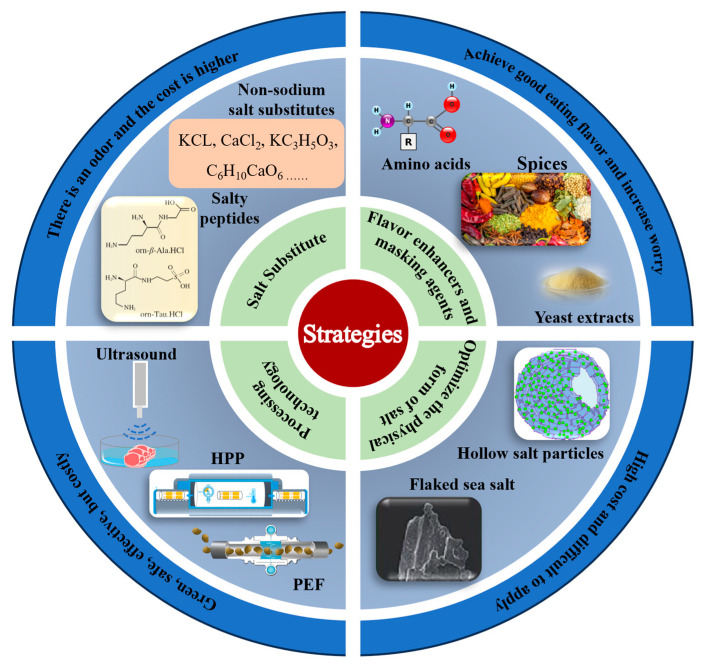
Salt reduction strategies in meat products.

Since ancient times, salt has been an indispensable seasoning in culinary practices, with its application and development being rich and diverse. Salt plays a crucial role in the quality of meat and meat products while also reducing microbial proliferation, thereby serving as a preservative and extending shelf life. However, excessive salt intake poses serious health risks to the human body. Therefore, salt in our diet is a “double-edged sword”, and achieving the balance between ensuring its basic processing characteristics and reducing intake has become a goal of research into healthy eating [1]. Figure 1 illustrates several mainstream strategies for salt reduction, each with its own challenges and limitations.

### 2.1. Salt Substitute

In everyday table salt, the main component is NaCl, while salt substitutes are substances with properties or functions similar to NaCl, which replace some or all of the sodium salts in table salt by substituting sodium ions, thereby achieving the purpose of salt reduction [2]. Currently, salt substitutes mainly consist of two categories: non-sodium salt substitutes and salty peptides. Non-sodium salt substitutes mainly include KCl, CaCl_2_, KC_3_H_5_O_3_, and C_6_H_10_CaO_6_, among others. Their functions and physicochemical properties are similar to sodium salt, so they are applied as salt substitutes in the production and development of low-salt meat products. Table 1 presents the current research on salt substitutes. Hu et al. employed the partial replacement of sodium chloride with potassium chloride in treating pork [27]. The results indicated beneficial effects on the physicochemical properties and gelation performance of pork myofibrillar proteins (MP). Non-sodium salt substitutes can effectively reduce the sodium content in meat products. However, due to the presence of some substances with off-flavors, they can negatively impact the taste of meat products and may not be accepted. Salty peptides are peptides with a saltiness intensity equivalent to or stronger than NaCl. They are extracted from foods such as beef broth, cheese, yeast extracts, and soy sauce extracts, or directly synthesized from amino acids. These peptides typically have a molecular weight ranging from 200 to 1500 Da, with most salty peptides being associated with fragments of glutamic acid and aspartic acid [2]. As a salt substitute, salty peptides possess functional properties highly similar to sodium salt and hold promising application value and development prospects. However, due to the high cost associated with the extraction or synthesis of salty peptides, large-scale production and application are currently unfeasible. In summary, salt substitutes have shown significant efficacy and great potential in reducing sodium content in meat products. However, current challenges include their impact on the flavor of meat products and the high cost of synthesis, which need to be addressed urgently.

### 2.2. Flavor Enhancers and Masking Agents

Flavor enhancers are substances that do not have a salty taste themselves but enhance the perception of saltiness when combined with salt, thereby intensifying the taste perception of salt by taste receptors. Flavor-masking agents, on the other hand, are substances that inhibit or block undesirable flavors such as bitterness in food. Food companies typically add both flavor enhancers and masking agents to food simultaneously to achieve a better taste profile. Based on the effects of taste–taste interactions, weak or near-threshold stimuli can lead to an overall enhancement of perception [33]. Consequently, recent studies have explored the use of salt-related flavor enhancers to amplify the perception of saltiness. Currently, major flavor enhancers and masking agents include amino acids, lactates, yeast extracts, nucleotides, phosphates, herbs, and spices, among others [30,31,38]. Guo et al. discovered that 0.8% lysine forms a fine network with more cross-links between myofibrils, significantly improving the water-holding capacity, texture, and sensory characteristics of reduced-salt ham [30]. Flavor enhancers and masking agents can effectively reduce sodium content and suppress off-flavors, but they also increase the number of additives in food. This can raise concerns among consumers and contradict the original intention of green food.

Could flavor enhancers or masking agents be used in combination with salt substitutes? This approach could effectively reduce off-flavors caused by metal ions in salt substitutes and also decrease the required amount of flavor enhancers or masking agents. Zheng et al. investigated the effects of L-lysine (Lys) and L-arginine (Arg) on the physicochemical and quality characteristics of reduced-sodium pork sausages (1.5% NaCl + 0.5% KCl) [32]. The results indicated that the combination of Lys/Arg with potassium chloride in low-salt sausages forms a dense and uniform three-dimensional network, thereby enhancing the water-holding capacity (WHC) and flavor of the sausages. Silva et al. conducted similar research, concluding that the combined use of amino acids and salt substitutes in producing low-sodium sausages is feasible while maintaining overall acceptability and sensory characteristics [31]. Therefore, the strategy of combining flavor enhancers or masking agents with salt substitutes in low-salt meat products is feasible, effectively addressing their respective shortcomings and deficiencies.

### 2.3. Optimize the Physical Form and Spatial Distribution of Salt

Major methods for optimizing the physical form of salt include reducing salt particle size and altering salt crystal structure [39]. Human taste receptors’ perception of saltiness is influenced by the size, internal structure, and shape of salt crystals. Therefore, optimizing the physical form of salt can enhance its dissolution and increase saltiness perception. Chen et al. prepared hollow salt particles (~10 μm) based on Quillaja saponin (QS) through spray drying and used them as solid carriers to enhance sensory aroma and reduce sodium intake [33]. Minter developed hollow salt microspheres based on amphiphilic biopolymers (such as gum Arabic), which can reduce salt content by 25–50% in certain applications (such as savory snacks) [40]. These hollow microspheres, with their high specific surface area, small size, and good free-flowing properties, may increase the accessibility or efficiency of sodium chloride to the target receptors on taste buds, thereby enhancing the perception of saltiness [33]. This strategy of optimizing the physical form of salt can effectively reduce the sodium content in meat products while ensuring that the perception of saltiness remains unaffected. However, the current approach may lead to higher production costs, reducing consumer willingness to purchase and diminishing product competitiveness. Additionally, although this strategy maintains the perception of saltiness, its impact on the processing performance of meat products (such as tenderness, WHC, and shelf life) has not been thoroughly studied. Additionally, research shown that the spatial distribution of salt in products greatly influences saltiness perception. Noort et al. proposed that altering the spatial distribution of salt, such as by making it evenly distributed, can increase saltiness perception [34]. This strategy is controversial. (1) Without reducing the total amount of sodium salt in the food, changing the spatial distribution of salt only increases the perception of saltiness by the human receptors, failing to achieve the goal of salt reduction. (2) While reducing the total amount of sodium salt in the food, changing the spatial distribution of salt makes it difficult to ensure that the quality of the food is not affected; therefore, achieving the goal of salt reduction by altering the spatial distribution of salt without compromising quality presents a significant challenge.

### 2.4. Non-Thermal Processing Technology

Currently, various novel non-thermal technologies are applied in food processing and manufacturing, such as ultrasound, high pressure, pulsed electric fields, etc. These technologies are widely researched due to their green, safe, and reliable nature, as well as their beneficial effects on low-salt food products. High-pressure processing (HPP) is a non-thermal processing technology that utilizes pressures ranging from 100 to 1000 MPa to achieve microbial inactivation, enzyme inactivation, and the improvement of meat quality, as well as the rapid freezing/thawing of food [41,42]. HPP technology holds great potential for the development and application of low-salt meat products. However, its widespread adoption and practical application are hindered by the relatively high costs associated with HPP equipment and maintenance expenses. Pulsed electric field (PEF) technology is an environmentally friendly technique where an electric field with intensities ranging from 0.1 to 100 kV is applied to meat and meat products between two electrodes. This facilitates the diffusion of NaCl within the muscle, thereby reducing the amount of salt used [37]. However, similar to the obstacles faced by HPP technology, PEF technology has not been widely applied in practice.

Numerous studies have shown that ultrasound can promote the uniform distribution of salt and shorten the curing time of meat products through cavitation and mechanical effects while reducing sodium salt usage. Additionally, ultrasound improves the WHC, tenderness, flavor, and protein functionality of low-salt meat products, enhancing their overall quality [11,35,43]. The effects of ultrasound on the quality characteristics of low-salt meat products and its mechanisms, as well as its advantages and disadvantages, will be reviewed in detail in the next section.

## 3. Effect of Ultrasound on Quality Characteristics of Low-Salt Meat Products

### 3.1. Texture

**Figure 2:**
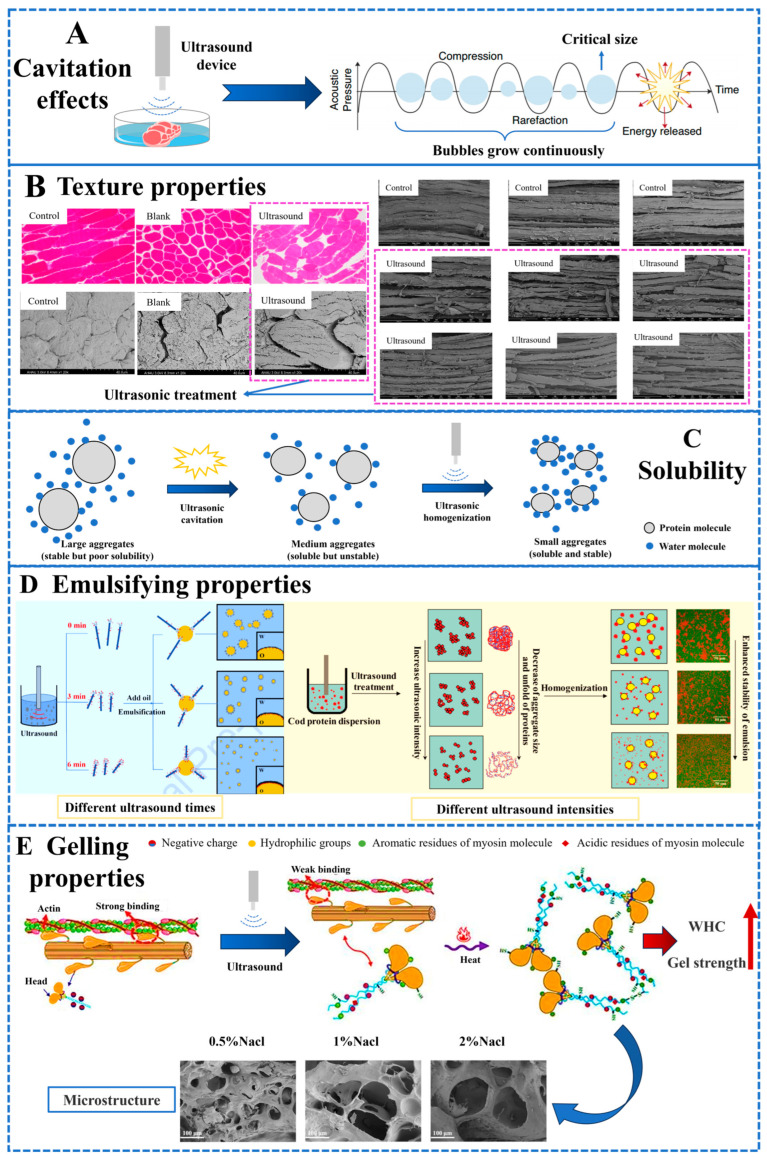
Principle of ultrasonic cavitation effect (**A**), muscle microstructure after ultrasound treatment at different angles (**B**), potential mechanisms by which ultrasound improves the solubility (**C**), emulsification properties (**D**), and gelling properties (**E**) of meat MP. Adapted from Zheng et al., 2024 [10], Gómez-Salazar et al., 2021 [11], Xiong et al., 2020 [46], Bai et al., 2023 [14], Shi et al., 2022 [48], Lin et al., 2020 [56], Ma et al., 2019 [57], Tang et al., 2021 [58].

The excellent texture properties of low-salt meat products directly influence consumers’ sensory experiences during consumption, with tenderness being a key aspect of chewing feedback when eating [44]. Typically, during the processing of meat products, salt interacts with salt-soluble proteins in the muscle, promoting their dissolution. This allows the muscle tissue to bind more water, increasing tenderness and WHC [1]. Therefore, maintaining good textural properties in low-salt meat products has become a focal point of recent research. Research has indicated that the ultrasound treatment of low-salt meat products helps improve tenderness [9,10,17]. As shown in Table 2, Shi et al. utilized ultrasound (5.6 W/cm^2^, 5 min) to assist in the tenderization of chicken breast meat brined with sodium alginate, and the results showed that this treatment significantly reduced the shear force values of the meat, thereby optimizing and improving the tenderness of the chicken breast meat [45]. Xiong et al. investigated the effects of ultrasound-assisted sodium bicarbonate curing on the curing efficiency and tenderness of chicken meat [46]. The results indicated that ultrasound-assisted pickling significantly improved the tenderness and WHC of chicken meat. As shown in Figure 2A,B, ultrasound could disrupt the muscle fiber structure through cavitation effects while promoting the penetration of sodium chloride into the meat. On one hand, this resulted in more structural protein degradation and dissolution of salt-soluble proteins. On the other hand, it helped to widen the gaps between muscle fiber bundles, allowing more water to be retained and improving the textural properties of low-salt meat products [1,17].

**Table 2:** Effect of ultrasound on quality, myofibrillar proteins, and gelation properties of low-salt meat products.

Sources	Treatment Parameters	Texture	WHC	Structure	Flavor	Permeability	Proteins Properties	Gelation Properties	Author
Tuna	4%, 6%, 8% NaCl + US (40 kHz, 840 W)	√	√	—	—	√	—	—	[47]
Pork	2% NaCl + US (37 kHz, 22 W/cm^2^)	×	×	—	—	√	—	—	[24]
Chicken	6% NaCl and 2% sodium bicarbonate + US (20 kHz, 300 W)	√	√	√	—	√	√	—	[46]
Chicken	NaCl + PA + US (15.6 W/cm^2^)	√	√	√	—	—	√	√	[45]
Sea bass	5% NaCl + US (20.5 kHz, 300 W)	√	√	√	√	√	—	—	[14]
Chicken	2% Phosphate + US (40 kHz, 300 W)	√	√	√	√	√	—	—	[15]
Beef	0.15% L-histidine + US (20 kHz, 300 W)	√	√	√	—	—	√	√	[48]
Beef	6% NaCl + US (20 kHz, 2.39, 6.23, 11.32 and 20.96 W/cm^2^)	—	—	√	√	—	√	—	[49]
Chicken	4% NaCl + 4% KCl + US	√	√	—	—	√	—	—	[50]
Beef	CaCl_2_ + US (40 kHz)	√	√	√	—	√	—	—	[51]
Dry-cured meat	Ultrasound treatment	√	√	√	√	√	√	√	[52]
Beef	US (20 kHz, 600 W)	—	—	—	√	√	√	—	[53]
Lamb	US (26 kHz, 1 W/cm^2^)	—	√	—	—	—	—	—	[54]
Pork	US (20 kHz, 600 W)	—	—	—	—	√	—	—	[55]

Note: “√” indicates the quality was improved; “×” indicates the quality was not improved; “—” indicates that the quality was not researched.

The WHC of meat products significantly impacts their economic value and eating quality, serving as a crucial indicator for assessing the textural characteristics of meat products [14]. Generally, in low-salt meat products, the reduction in salt content leads to a decrease in the binding capacity between meat and water molecules, resulting in increased loss of juices during cooking. Tong et al. found that ultrasound-assisted curing mechanically disrupted the muscle fiber structure, increased the space between muscle fiber bundles, facilitated water migration and uniform distribution, and improved the curing rate, WHC, and tenderness of the meat [15]. Shi et al. utilized ultrasound-assisted L-lysine curing of beef, and compared with other treatment groups, the ultrasound group exhibited the highest WHC [48]. On one hand, as previously described, the cavitation effect of ultrasound enlarged the gaps between muscle cells and muscle bundles, leading to more relaxed muscle fibers and creating more space for water binding [17]. On the other hand, ultrasound promoted the penetration and uniform distribution of salt within the muscle, altering the spatial structure of more proteins. The aggregation of sodium ions increased the net positive charge carried by protein molecules and enhanced electrostatic repulsion between molecules, thereby improving WHC [1].

However, improper ultrasound application may degrade the texture of meat products. Leong et al. [22] and Gallo et al. [23] found that high-intensity or high-power ultrasound can cause severe structural damage to muscle fibers and protein denaturation, leading to increased toughness, firmer texture, and reduced WHC. Therefore, it is crucial to identify suitable ultrasound parameters (such as frequency, power, and duration) for different low-salt meat products to effectively improve their textural properties [1].

### 3.2. Flavor

**Figure 3:**
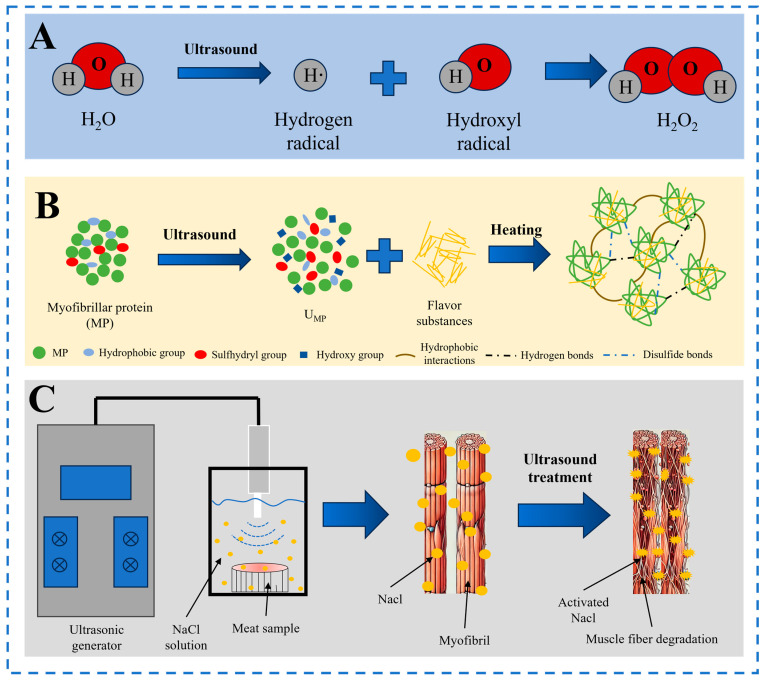
Water molecules are decomposed into highly reactive free radicals after ultrasound treatment (**A**). Ultrasound promotes the production of volatile flavor compounds (**B**). Ultrasound disrupts the structure of myofibrils and activates the brine, accelerating the penetration of sodium chloride (**C**).

Flavor serves not only as a crucial indicator for assessing the eating quality of meat and meat products but also as a significant factor influencing consumer choices and purchase desires. The formation of flavor in meat products primarily occurs through two pathways: the breakdown and conversion of flavor precursors, and the degradation of volatile flavor compounds [14]. Flavor precursors mainly comprise amino acids and fatty acids, which undergo a series of reactions during cooking processes. For instance, proteins and lipids are hydrolyzed to produce free amino acids and free fatty acids, which, upon undergoing Maillard reactions with reducing sugars, generate a series of aromatic compounds [49]. Studies have found that, compared to static marination, ultrasound-assisted marination promotes the degradation and oxidation of beef proteins and lipids [49]. As shown in Figure 3A, the cavitation effect of ultrasound causes water molecules to break down, generating highly reactive hydroxyl radicals, leading to the oxidation of lipids and proteins in meat [59]. The oxidation of proteins and lipids contributes to the formation of aldehyde and ketone flavor compounds [60]. Meanwhile, volatile flavor compounds are released during heating through degradation, including aldehydes, alcohols, acids, ketones, and esters as well as nitrogen- and sulfur-containing compounds. Taste substances mainly consist of amino acids, nucleotides, and sugars [61].

At the molecular level, muscle proteins can bind with flavor compounds through chemical forces such as hydrogen bonds, van der Waals forces, and hydrophobic interactions (Figure 3B) [62]. As previously mentioned, ultrasonic treatment induces active radicals by dissociating water molecules, which alters the structure of MP and the intermolecular forces. This, in turn, affects the binding capacity between MP and flavor compounds [59,60]. As shown in Table 2, Bai et al. found that ultrasound-assisted marination significantly increased protein degradation and total free amino acid levels as well as the relative levels of aldehydes and esters, among other volatile flavor compounds, in seabass [14]. Tong et al. [15] and Kang et al. [49] explored the effects of ultrasound-assisted marination on protein and lipid oxidation in meat products. Their results indicated that, compared to static marination, ultrasound treatment significantly increased TBARS values, carbonyls, and total sulfhydryl content (*p* < 0.05). These findings collectively suggest that ultrasound treatment can enhance protein oxidation, degradation, and lipid oxidation in meat and meat products, further promoting the formation and release of desirable flavors in low-salt meat products. However, some studies have found that high-intensity/high-power ultrasound can lead to excessive protein oxidation in marinated meat, potentially resulting in negative effects on flavor due to oxidation by-products [24,25]. Similarly, while the cavitation effect of ultrasound generates reactive free radicals from molecules and water, excessively high intensity or prolonged exposure can lead to the formation of highly oxidative H_2_O_2_ (Figure 3A), causing strong oxidation of lipids and proteins in the meat, thereby promoting chain reactions such as lipid oxidation or protein hydrolysis [53,59]. In turn, these reactions can affect derived characteristics, such as reversible or irreversible changes in existing protein structures or the generation of undesirable flavors [45,63]. Therefore, it is essential to determine the appropriate ultrasonic parameters for different low-salt meat products to maximize the potential and advantages of ultrasonic technology in enhancing their quality.

### 3.3. Ion Permeability

High-salt curing of meat products relies on the high ion concentration of the external environment to achieve complete curing. However, products cured through traditional methods often suffer from issues such as prolonged curing time, uneven distribution of saltiness, significant flavor disparities, and unstable quality [46]. In recent years, research has shown that the application of ultrasound technology in processing low-salt meat products helps to shorten the curing time, promote uniform distribution of salt, and reduce the amount of salt used [14,24]. Currently, most studies on the use of ultrasound during the marination of meat and meat products have only addressed mass transfer kinetics [49,64] or technical characteristics [63,65]. The novelty of our research lies in the combined assessment of the effects of ultrasound application on salt diffusion and myofibrillar proteins, such as meat tenderness, water-holding capacity, muscle fiber structure, and protein denaturation. This combined assessment is crucial for establishing appropriate conditions to maximize the potential and advantages of ultrasound technology in improving the quality of meat products.

Inguglia et al. investigated the effect of high-power ultrasound (25, 45, 130 kHz) on the brining of chicken breast meat [50]. The results demonstrate that ultrasound-assisted brining significantly increases the salt content of chicken meat compared to the control group (NaCl content less than 0.05), which facilitates the brining process and reduces sodium chloride content. Wan et al. explored the impact of ultrasound-assisted calcium chloride immersion on the tenderness of beef semitendinosus muscle [51]. They found that low-intensity and low-frequency ultrasound-assisted calcium chloride immersion promotes the transmembrane transport of calcium ions through the calpain system, thereby improving meat tenderness. When investigating the ultrasound-assisted brining of tuna with different NaCl concentrations, Yao et al. found that the application of ultrasound increased the effective diffusion coefficient (De) of salt during the brining process from 402.8% to 653.21% [47]. Additionally, the highest De was observed at a 5% salt concentration, indicating that the application of ultrasound can improve the uniformity of salt distribution. In summary, ultrasound disrupts the structure of myofibrils through cavitation effects, increasing the inter-fiber space and the degree of actin dissociation, thereby creating channels [11]. On the other hand, as shown in Figure 3C, ultrasound can activate brine and accelerate the movement of salt ions during the marination process [16]. Through these mechanisms, ultrasound quickly achieves a state of equilibrium in the concentration gradient between brine and meat tissue [66], thereby promoting the rate of salt penetration and its uniform distribution [14,17]. Although ultrasound treatment can shorten the marination time by 2.5 to 3 times and increase the permeability of muscle fibers to brine components, damage to myofibrils can be observed [54]. Therefore, it is necessary to select the optimal ultrasound marination parameters.

## 4. Effect of Ultrasound on Properties of Proteins and Gel

### 4.1. Solubility

In meat and meat products, MP is a salt-soluble protein that requires a certain ion strength salt solution to dissolve [2]. The reduction in salt addition in low-salt meat products will inevitably affect the solubility of MP to some extent. Solubility is one of the important physicochemical indicators of MP and is also one of the factors affecting protein functionality. It not only affects the foaming and emulsifying properties of MP but also directly reflects the denaturation and aggregation degree of proteins. According to Figure 2C, ultrasound, through its cavitation effect and mechanical action, promotes the transformation of proteins from stable insoluble aggregates to stable soluble complexes, exposing more hydrophilic groups, thereby enhancing the solubility of proteins [10].

In recent years, studies have shown that the solubility of MP in low-salt meat products treated with ultrasound technology has been significantly improved (Table 2). Wang et al. found that with increasing ultrasound time, the protein solubility increased significantly from 6.55% to 9.02% within 6 min (*p* < 0.05), while extending the ultrasound treatment time to 9–15 min resulted in a gradual decrease in MP solubility [67]. The decrease in MP solubility observed between 9 and 15 min can be explained as follows: protein molecules unfold and expand, exposing more hydrophobic and thiol groups, leading to changes in the aggregation of large molecules. Shi et al. found that ultrasound induced the initial dissociation of myosin through physical effects, disrupting the natural interactions of protein aggregation, and enhancing protein solubility [48]. In summary, ultrasound technology contributes to the improvement of MP solubility in muscles. However, due to variations in ultrasound equipment, power, frequency, duration, and meat samples, the effectiveness of enhancing MP solubility in muscles may vary.

### 4.2. Emulsifying Properties

Under low-salt conditions, the emulsifying properties of meat products, mainly reflected in the emulsifying activity index (EAI) and emulsion stability index (ESI) of MP, are affected. The EAI represents the ability of proteins to adsorb at the interface between the oil and water phases during the formation of an emulsion, with higher EAI values indicating a stronger adsorption capacity of MP at the interface. The ESI, on the other hand, indicates the ability of MP to maintain the stability of the emulsion dispersion system after a certain period of storage [56].

Research has shown that ultrasound technology is beneficial for improving the emulsifying properties of meat product MP (Table 2). As depicted in Figure 2D, the application of ultrasound technology to enhance the EAI and ESI of MP in low-salt meat products has become a focal point of research for domestic and international scholars. Amiri et al. investigated the effects of high-intensity ultrasound (20 kHz) at different times (10, 20, and 30 min) and powers (100 and 300 W) on the physicochemical properties of beef MP [19]. They found that with increasing ultrasound time and power, the EAI significantly increased, with the highest emulsifying activity observed at an ultrasound power of 300 W. This increase in EAI may be attributed to the alteration of MP structure by ultrasound treatment, resulting in a higher surface area-to-volume ratio, thereby allowing more proteins to participate in the formation of the interface layer and consequently enhancing emulsifying activity. Additionally, the cavitation and mechanical effects of ultrasound led to partial denaturation and structural disruption of MP, as well as changes in protein particle size distribution and surface area, thus increasing the adsorption capacity of MP at the water–oil interface. Consistent with these findings, Amiri et al. also observed a similar trend in the changes of ESI, which increased mainly due to the cavitation effect of ultrasound [19]. Similarly, Li et al. concluded that under the influence of higher ultrasound intensity (20 kHz, 450 W) for 6 min, the emulsifying performance of MP was significantly improved [68]. Furthermore, Ma et al. observed uniform microstructures and smaller oil droplet sizes in stable emulsions of cod fish protein treated with high-intensity ultrasound, indicating a significant improvement in aggregation effects [57]. In conclusion, ultrasound treatment can effectively enhance the emulsifying properties of MP in low-salt meat products. Similarly, due to variations in ultrasound equipment, power, frequency, duration, and meat samples, the improvement effects on the emulsification properties of muscle MP will also differ.

### 4.3. Strength and WHC of Gel

Gel strength refers to the product of the breaking force (in grams) and the breaking distance (in millimeters) when a gel breaks. A higher product indicates better gel strength and quality [56]. Tang and Yongsawatdigul found that the texture characteristics and color of fish mince gel with 0.5% NaCl improved with increasing ultrasound intensity [58]. Additionally, Shi et al. also concluded that ultrasound promotes the formation of thermally stable gel protein structures [48]. As shown in Figure 2E, during the gel formation process, MP form a stable three-dimensional network structure, which traps water within the structure. Therefore, the WHC of the gel reflects the ability of muscle proteins to retain moisture in their three-dimensional structure, and also reflects the spatial structure of muscle MP gel [69]. Zhang et al. found that the WHC of shrimp mince gel increased with increasing ultrasound power or time [70]. This increase may be attributed to the promotion of interactions between proteins and water by ultrasound treatment, leading to the partial unfolding of protein chains, making polar residues more accessible to capture water within the gel [20]. In addition, Zhou et al. found that ultrasound affects the gel properties by influencing protein molecules and the interaction between proteins and water molecules, thereby increasing the gel’s WHC [21].

However, variations in the frequency, intensity, and duration of ultrasound treatment can differently affect gel formation and structure. Zhang et al. found that with increasing ultrasound power, the gel strength of shrimp mince initially increased and then decreased [70]. The ultrasound group (360 W, 20 min) exhibited the highest gel strength, which was 54.02% higher than that of the non-ultrasound pretreatment group. The trend of gel strength variation with different ultrasound treatment times was consistent with that of different ultrasound powers and consistently higher than that of the control group. Yu et al. found that low-power and short-duration ultrasound treatment might disrupt hydrogen bonds in the secondary structure of protein gels, leading to a decrease in the gel’s WHC [71]. This could also be due to the fact that low-power, short-duration ultrasound treatment causes less protein chain unfolding and aggregation, weakening the gel structure and reducing its water absorption capacity [72]. Therefore, appropriate ultrasound treatment has a positive impact on the WHC of MP gels in low-salt meat products.

### 4.4. Microstructure

The microstructure of gels is of great significance for evaluating the gel formation process and its physical properties. Zhang et al. found that with the extension of ultrasound pretreatment time, the gel structure of low-salt South American white shrimp became denser and slightly rougher after 20 min [70]. Except for the ultrasound pretreatment group (600 W, 20 min), other pretreatment groups exhibited relatively dense structures, and the gel network was most uniform and dense in the ultrasound group (360 W, 20 min). This is because the degree of gel cross-linking was maximal, thus forming new proteins through the binding of free amino acids and proteins and promoting the aggregation of proteins. However, the gel network structure of the sample treated with ultrasound (600 W, 20 min) was loose and disordered, possibly due to the destruction of the gel microstructure by high-power ultrasound, leading to protein denaturation, reduced protein solubility, the formation of insoluble aggregates, and the formation of rough gel networks [73]. Tang and Yongsawatdigul, using scanning electron microscopy to observe the effects of high-intensity ultrasound on the gel properties of fish mince with low NaCl content, found that at 0.5% NaCl, the formed gel network was more regular, with an increased number of pores and a significantly improved WHC [58]. However, at 1% and 2% NaCl concentrations, high-intensity ultrasound treatment led to poorer gel quality (as shown in Figure 2E). Therefore, appropriate ultrasound pretreatment can promote the formation of well-structured gels in low-salt meat products.

## 5. Conclusions

Ultrasound technology, as a novel, green, safe, and reliable non-thermal processing technology, offers the following advantages in improving low-salt meat products. (1) Ultrasound, utilizing its cavitation and mechanical effects, effectively shortens the marination time of low-salt meat products and promotes the even distribution of salt, thus reducing the amount of salt used while ensuring the high quality of low-salt meat products. (2) Ultrasound technology improves the WHC of low-salt meat products, enhances meat tenderness, and promotes the formation and release of desirable flavors. (3) Ultrasound, through its cavitation and mechanical effects, facilitates the transition of proteins from stable insoluble aggregates to stable soluble complexes, exposing more hydrophilic groups and, thus, enhancing protein solubility. At the same time, ultrasound promotes a greater number of proteins to participate in the formation of interfacial layers, thereby increasing emulsifying activity. Furthermore, ultrasound treatment promotes the interaction between proteins and water, leading to partial unfolding of protein chains, which allows polar residues to more readily capture water in the gel, thereby improving the water-holding capacity of the gel. These effects will contribute to the formation of high-quality low-salt meat products. However, variations in the frequency, intensity, and duration of ultrasound treatment result in different effects on the quality improvement of low-salt meat products. Therefore, it is essential to identify suitable ultrasound parameters for different low-salt meat products to maximize the potential and advantages of ultrasound technology in enhancing their quality.

## Figures and Tables

**Table 1:** Research on strategies to reduce sodium chloride (NaCl) in different products.

Strategy	Product	Method	Results	References
Salt substitute	Porcine MP	70% NaCl + 30% KCl 70% NaCl + 20% KCl + 10% other components	The partial replacement of NaCl by KCl combined with other components improves the physicochemical and gel properties of MPs.	[5]
	Dry cured bacon	45% NaCl + 25% KCl + 20% CaCl_2_ + 10% MgCl_2_ 30% NaCl + 50% KCl + 15% CaCl_2_ + 5% MgCl_2_	Promoting the hydrolysis of dry-cured lacón and lipids, which, in turn, promotes the production of flavor substances.	[28]
	Ham	70% NaCl + 30% KCl 60% NaCl + 40% KCl	The NaCl content of ham can be reduced by 30% and 40%, respectively, while maintaining good sensory acceptance.	[29]
Flavor enhancers and masking agents	Salt-reduced ham	1.25% NaCl + 0.6% Lys 1.25% NaCl + 0.8% Lys	The addition of 0.8% lysine significantly improves the WHC, texture properties, and sensory attributes of ham.	[30]
	Bologna-type sausage	1% NaCl + 1.5% KCl + 1% arginine + 0.2% histidine	It is feasible to produce bologna sausages with reduced sodium content (approximately 40%) while maintaining overall acceptability and sensory characteristics.	[31]
	pork sausages	0.6% lysine + 0.5% KCl 0.6% arginine + 0.5% KCl	The combination of Lys/Arg with potassium chloride in low-salt sausages forms a dense and uniform three-dimensional network, thereby enhancing the water-holding capacity and flavor of the sausages.	[32]
Optimize the physical form and spatial distribution of salt	Venison	4%, 6% or 8% NaCl	Dry-cured venison with lower NaCl content showed higher proteolysis and reduced textural properties.	[7]
	Hollow salt particles	Perilla saponin-based hollow salt particles developed by spray drying (~10 μm)	Improving flavor performance by reducing sodium intake.	[33]
	Bread	A strategy using uneven spatial distribution of Na	The Na content in bread can be significantly reduced without losing saltiness intensity and without the use of sodium substitutes or taste or aroma additives.	[34]
Non-thermal processing technology	Wooden breast meat	1% NaCl + US 2% NaCl + US	Ultrasound can improve the gel properties of meat and has the potential to produce low-salt gel meat products.	[35]
	Emulsified beef sausage	1.4% NaCl + 200 MPa HHP	High-pressure treatment at 200 MPa achieves texture and sensory attributes that consumers prefer.	[36]
	Sea bass	5% NaCl + PEF 10% NaCl + PEF	PEF pretreatment can effectively shorten the brine curing time and increase the salt absorption to 77% while ensuring its even distribution in the muscle.	[37]
